# Supplementary material for: Are carbon emissions trading and green financial instruments synergistic? -Comprehensive quantitative research based on content analysis
Source: PLoS One. 2024 Mar 7;19(3):e0298601. doi: 10.1371/journal.pone.0298601 (PMC10919601; doi:10.1371/journal.pone.0298601)
Supplement: S1 Data — (ZIP) [file pone.0298601.s001.zip › Data/地级市Z_F工作报告.zip!/╔╜╢1⁄2/╤╠╠¿╩╨/└┤╫╘╣1⁄2╓┌║┼í╛┬φ┐╦╩2╛▌═°í┐_2016.docx]

各位代表：

现在，我代表市人民政府向大会报告工作，请予审议，并请市政协委员和其他列席会议的同志提出意见。

一、“十二五”时期工作回顾

“十二五”是我市发展史上极不平凡的五年，是砥砺奋进的五年，也是成就辉煌的五年。五年来，面对复杂多变的国内外经济形势和艰巨繁重的改革发展稳定任务，全市上下在中共烟台市委的坚强领导下，认真贯彻执行中央、省各项决策部署，开拓创新、攻坚克难，经济社会持续平稳健康发展。“十二五”规划安排的30项主要指标中，有12项约束性指标和16项预期性指标超额完成，2项预期性指标接近目标值，“十二五”胜利收官。

过去的五年，全市主要经济指标实现“三个明显高于”，发展质量效益达到新水平。五年来，在经济下行压力持续加大的形势下，我市主要经济指标增速始终明显高于全国、全省和首批14个沿海开放城市平均水平。全市生产总值年均增长10%，连续突破5000亿元、6000亿元，2015年达到6446.1亿元，人均生产总值是“十一五”末的1.6倍、达到中高收入国家水平。一般公共预算收入连续跨过300亿元、400亿元、500亿元，2015年达到542.7亿元，年均增长17.9%。固定资产投资和社会消费品零售总额分别达到4667.1亿元和2679.5亿元，年均分别增长18.5%和13.9%。产业结构调整深入推进，三次产业之比由2010年的7.7：58.9：33.4调整为6.8：51.6：41.6。农业现代化水平不断提高，粮食生产实现“十三连丰”，“海上粮仓”建设全面启动，新型农业经营主体健康发展，打造了东方海洋、鲁花、龙大、民和牧业、双塔食品等一批知名品牌、放心品牌，全市农产品出口占到全国的1/20。工业实力不断增强，规模以上工业主营业务收入、利税和利润年均分别增长10.9%、9.1%和8.8%，2015年分别达到15351亿元、1460亿元和1101亿元，主营业务收入过百亿元企业由12家增到16家，形成了汽车制造等5个千亿级产业集群，是全省唯一连续10年完成节能减排目标的市。万华工业园、张裕国际葡萄酒城、上汽通用东岳新车型等重大项目建设投运，富士康(烟台)工业园、南山集团、招金集团、中集来福士、杰瑞集团、绿叶集团等骨干企业纷纷进军新领域。服务业升级步伐不断加快，金融、物流、旅游、养老、文化、体育等产业蓬勃发展，万达广场、中粮大悦城等商贸综合体建成投用。华海财险组建运营，恒丰银行资产突破万亿元，全市金融机构本外币存贷款余额分别达到7030亿元和4470亿元。累计接待海内外游客2.48亿人次，实现旅游收入2720亿元，国内首艘全资自主经营管理的邮轮“中华泰山”号顺利运营。引进20多个大型养老项目，成为全国养老服务产业试点市。新认定山东名牌产品242个、服务名牌87个，一批烟台企业、烟台产品正以崭新面貌闪亮中国、走向世界。

过去的五年，全市改革开放创新实现“三个纵深推进”，发展动能开始出现新转换。各领域改革纵深推进，实施国家、省改革试点106项，市级行政审批事项精简49.8%、行政权力事项压减48.7%，国企、农村、医药卫生、教育、政府机构、事业单位以及财政、科技、文化、价格等领域改革迈出坚实步伐，农信社银行化改革提前完成。对外开放纵深推进，累计使用外资80.5亿美元，是“十一五”时期的1.6倍，新增世界500强投资企业15家、项目24个，总数分别达到94家和138个。2015年进出口总额493.9亿美元。新缔结7个国际友好城市，总数达到24个。创新驱动纵深推进，引进了3个国家级科研院所，新增国家级产业技术创新战略联盟、工程技术研究中心、企业技术中心11家，发展高新技术企业146家，申请发明专利1.8万件，获得国家科技奖励19项，引进人才15.5万人，被评为全国科技进步先进市、国家知识产权示范城市。烟台开发区综合实力稳居全国219个国家级开发区第6位，保税港区进出口规模上升到全国14个保税港区第1位，烟台高新区、招远开发区晋升为国家级，昆嵛山保护区生态功能进一步增强，东部新区拉开发展框架，5个省级开发区进入全省30强。

过去的五年，全市基础设施、城乡面貌、生态环境实现“三个较大改善”，统筹城乡发展取得新突破。基础设施实现较大改善，青烟威荣城际铁路通车运行，烟台蓬莱国际机场建成通航，胶东调水工程贯通通水，龙烟铁路开工建设，烟台港西港区40万吨矿石码头等一批港口工程建成投运，港口货物年吞吐量达到3.3亿吨，稳居国内十大沿海港口之列。丁字湾跨海大桥、长岛南北长山大桥竣工通车，蓬莱至长岛实现跨海供水。海阳核电一期顺利推进，华电莱州电厂一期投入运营。新建成4条高速公路，新增农村公路4224公里，公路通车里程达到1.88万公里。城乡面貌实现较大改善，城镇化率由55.3%提升到60.4%。《烟台市城市总体规划(2011-2020年)》获国务院批复。市区城建投入1500多亿元，实施了白石等10多个区片改造和700多个重点项目，建成区面积由265平方公里扩展到316平方公里。

全市新建扩建生活垃圾发电项目5个、污水处理厂7座，新增集中供热面积851万平方米、天然气用户42万户。15个镇成为全国重点镇。建成新型社区346个，改造农房30.8万户，农村自来水普及率达到96%，生活垃圾一体化管理实现全覆盖。对16座大中型和321座小型水库进行了除险加固。生态环境实现较大改善，重点河流监测断面水质达标率稳定在85%以上，门楼水库总氮浓度均值下降40%以上。造林绿化116万亩，被评为全国绿化模范城市。空气质量稳居全省第二位。

过去的五年，全市社会事业、民生保障、综治维稳实现“三个巩固提升”，社会和谐水平达到新高度。社会事业持续巩固提升，新建扩建学校、幼儿园617所，所有县市区全部通过国家义务教育发展基本均衡验收。新增医疗卫生机构236所。全社会累计爱心捐款15.3亿元，救助困难群众268万人次。深入挖掘胶东红色文化，圆满承办第十届中国艺术节，被评为全国文化先进市。烟台籍运动员在重大赛事中屡创佳绩。民生保障持续巩固提升，城镇和农村居民人均可支配收入分别达到35907元、15540元，年均分别增长10.6%和12.8%。五年新增城镇就业88.6万人，农村劳动力转移就业19.2万人，城镇登记失业率控制在3.25%以内。整合了城乡居民养老保险、医疗保险，各项社会保障待遇稳步提高。创设了“89000”民生服务平台。筹建保障房2.7万套，改造棚户区7.66万户。综治维稳持续巩固提升，信访工作扎实有效，安全生产、食药安全、社会治安形势总体平稳。“法德共进”经验在全省推广。人口计生工作保持先进水平。对口支援西藏、新疆等工作取得新成效。国防动员、民兵预备役、人民防空、双拥共建工作深入开展。老龄、妇女、儿童、残疾人、红十字事业健康发展，统计、史志、档案、仲裁、机关事务、新闻出版、广播电视、民族宗教和气象、地震等工作取得新成绩。

各位代表，2015年是“十二五”收官之年，我们承接长期积淀的良好基础，围绕实现年度和五年的“双目标”，坚持工作重心下移，脚踏实地推进各项工作。着力强化稳增长措施。出台经济转型升级10条、稳外贸16条、工业三年行动计划等一系列政策措施，引进设立了城镇化、健康养老等34支规模达340亿元的各类基金。全市生产总值增长8.4%，第三产业增加值占比提高1.7个百分点，一般公共预算收入增长10.7%，实际使用外资增长8.3%，一般贸易出口增长13.8%，进出口总额占全省1/5以上。着力培育发展新动能。按领先型、支柱型、追赶型、成长型、转移型“五种类型”引导产业转型升级，重点扶持发展系统重要性产业，高新技术产业产值占比达到41.1%，工业36个行业大类中有29个增加值增长，七个战略性新兴产业总产值增长11.5%。新增上市公司4家，总数达到38家，居全省首位，另有2家已通过审核即将上市，75家企业在“新三板”或区域性股权交易市场挂牌。成功组建海洋产权交易中心。电子商务交易额增长35.8%。获批国家应急产业基地和国家海洋高技术产业基地。着力营造开放新优势。中韩(烟台)产业园成为中韩自贸协定框架下中韩两国共建产业园区，与韩中(新万金)产业园建立“两国双园”合作模式，对韩贸易110.8亿美元、占全省1/3以上。与“一带一路”沿线国家全部建立经贸往来，全市境外实际投资增长37%。成功举办东亚经济交流推进机构第五届总会。着力加强涉企服务。建立“三清单一平台”，砍掉了一批办事关卡，完善企业联系人制度和政银企联席会议制度，推广自贸区可复制政策，集中解决要素保障等六个方面问题，取消、停征、免征、降低81项行政事业性收费，减轻企业负担2.5亿元，新登记市场主体增长97.3%，其中企业增长66.8%。着力办好惠民实事。12件为民服务实事全部完成。4.3万人实现脱贫。城镇和农村居民人均可支配收入分别增长7.8%和8.9%。市区空气优良天数达到304天，比上年增加24天。我市被评为国家级海洋生态文明建设示范区。烟台格罗宁根大学在中荷两国元首见证下签署办学协议。

各位代表，过去五年，面对深刻变化的经济形势，能够取得这样的成就实属不易，创造的财富影响深远，积累的经验弥足珍贵。走过“十二五”，我们深切感到，正是坚持理念上与时俱进、目标上高点定位、措施上大胆探索，才引领了烟台经济行稳致远。特别是十八大以来，我们从抓“速度机遇”到抓“转型机遇”，确立了率先走在前列的战略定位，明确了“六抓六促”“六个持续”的工作思路，工作指导的重大转变激发了烟台发展新动力。全市生产总值上升到全国城市第20位，规模以上工业主营业务收入上升到第9位。走过“十二五”，我们深切感到，正是在机遇面前积极作为、在挑战面前直面应对、在困难面前矢志不移，才实现了烟台经济破难前进。抢抓“蓝黄”两区建设、扩大内需等重要机遇，启动了一批重大项目，拉开了“一极领先、多极崛起”格局。特别是面对经济下行的严峻形势，变挑战压力为转型升级动力，倒逼结构调整取得明显进展。走过“十二五”，我们深切感到，正是改革的不断深化、开放的纵深推进、创新的持续突破，才聚集起支撑烟台发展的正能量。着眼解决长期矛盾和困难的综合改革增强了动力与活力，推进产业、市场、企业、园区、城市“五个国际化”提升了开放型经济水平，各个领域的创新举措和成功实践培育出发展的新动能。走过“十二五”，我们深切感到，正是攻坚克难的魄力、坚韧不拔的毅力、众志成城的合力，才成就了烟台重点工作的新突破。全市上下万众一心，共同应对各类突发事件，合力维护发展大局，取得了全国文明城市“四连冠”、全国社会管理综合治理“六连冠”、国家历史文化名城等荣誉，结束了烟台无动车、无专属民用机场、无客水的历史，第三届亚沙会向世界展示了烟台风采。走过“十二五”，我们深切感到，正是保持了执政为民的理念、改善民生的担当、为民谋利的执着，才提升了烟台人民的幸福指数。坚持每年兴办一批为民服务实事，每年提高民生保障标准，民生支出占财政支出比重上升到77.2%，烟台幸福指数在多家专业机构评价中居全国前列。走过“十二五”，我们深切感到，正是践行群众路线、开展“三严三实”教育、持续改进作风，才促进了干部队伍素质的整体提升。政府系统不断加强自身建设，持续强党建、转作风、抓纪律、反腐败。深入推进依法行政，建立政府法律顾问制度，加强行政监察和审计监督，决策程序和行政行为更加规范。自觉接受市人大的法律监督和市政协的民主监督，累计办理市人大代表建议522件、政协提案2501件。这些在实践中积累的规律性认识，必将成为推动新一轮发展的宝贵财富。

各位代表，五年来我市取得的每项成绩，都是党中央国务院、省委省政府和市委正确领导的结果，是市人大、市政协和社会各界大力支持的结果，是全市上下努力拼搏的结果。在此，谨代表市人民政府，向全市各族人民，向市人大代表、政协委员，向各民主党派、工商联和无党派人士、人民团体，向驻烟人民解放军指战员和武警官兵，向中央省属驻烟单位，向各位老领导、老同志，向关心支持烟台发展的社会各界人士和海内外朋友们，表示衷心的感谢，并致以崇高的敬意！

各位代表，过去五年成就巨大，但我们也清醒地看到，我市经济社会发展还面临不少困难和问题。经济下行压力较大，新旧动能正在转换，培育新的经济增长点任务艰巨繁重；结构性问题仍然突出，创新驱动动力不足，部分行业企业生产经营困难；改革进入攻坚期，阻碍发展的体制机制亟待破除；财政收入增速放缓，刚性支出持续增加；公共服务与群众期望仍有一定差距，扶贫攻坚任务艰巨；环境保护与经济发展的矛盾仍然突出，安全生产方面还存在薄弱环节；部分政府工作人员执政为民的服务意识、依法行政的法治观念需要强化，在一些领域还存在不正之风和违纪违法问题。我们将坚持目标导向和问题导向，逐一化解矛盾、突破难题，为更好更快发展扫清障碍、铺路搭桥。

二、“十三五”时期的总体思路和目标任务

“十三五”时期是实现第一个百年奋斗目标、全面建成小康社会的决胜阶段。面向未来五年，我们编制了《烟台市国民经济和社会发展第十三个五年规划纲要(草案)》，《纲要(草案)》及《说明》已印发大会，请一并予以审议。“十三五”时期，政府工作的总体思路是：全面贯彻党的十八大和十八届三中、四中、五中全会精神，以邓小平理论、“三个代表”重要思想、科学发展观为指导，深入落实习近平总书记系列重要讲话和视察山东重要讲话、重要批示精神，按照“五位一体”总体布局和“四个全面”战略布局，牢固树立和贯彻落实创新、协调、绿色、开放、共享的发展理念，紧紧围绕率先走在前列的战略定位，瞄准“一二三四五”的总目标，以提高经济发展质量效益为中心，深入推进“六抓六促”“六个持续”，把握稳增长与调结构的平衡，着力做强实体经济，着力加强生态建设，着力保障改善民生，依靠改革的主动、开放的主动、创新的主动赢得发展的主动，确保决胜全面小康、率先走在前列。

决胜全面小康、率先走在前列，就要践行五大发展理念、开创科学发展新局面。坚持率先创新发展，实施全域创新，掀起技术、制度、管理、文化等各个方面的创新热潮，打造创新烟台、创业港城、创客家园、创富城市，努力建设国家创新型城市。突出技术创新，以高新技术支撑产业的未来。到2020年，高新技术产业产值占比达到43%，全社会研发投入占比达到3%以上。坚持率先协调发展，围绕提升中心城市首位度、打造价值中心，精心建设主城区，拓展东部蓝色经济和高新技术产业示范区，发展西部高端制造业和临港产业核心区，拉开“一城居中、两翼齐飞、融合发展”的市区空间布局。统筹推进区域开发建设，着力打造北部经济隆起带、南部经济崛起带、中部生态涵养带，构筑现代城市与富美乡村和谐相融的城乡空间布局。到2020年，城镇化率达到65%。坚持率先绿色发展，守护好“山海岛泉河”生命共同体，创建国家森林城市、水生态文明城市，打造生态美丽典范城市。到2020年，森林覆盖率达到42%以上，市区空气优良天数保持在310天以上。坚持率先开放发展，深入实施“五个国际化”，促进“引进来”与“走出去”双向突破，实现外贸“大进大出”向“优进优出”转变。到2020年，对外贸易达到630亿美元，五年使用外资100亿美元。坚持率先共享发展，健全共建共享机制，实现投资有效益、产品有市场、企业增利润、员工增收入、政府增税收。到2020年，城镇和农村居民人均可支配收入分别达到5万元、3万元以上，城镇登记失业率控制在3.5%以内，使发展成果更多转化为群众的获得感和满意度。

决胜全面小康、率先走在前列，就要突破重点、带动全局。在增创政策新优势方面，推进中韩(烟台)产业园“两国双园”合作，参与中日韩地方经济合作，争取建设山东自贸区烟台片区。在培育经济新引擎方面，发展临港综合产业区，推进山东半岛自主创新示范区建设，打造海洋产业高地，建设山东省区域性基金管理中心。在壮大产业新集群方面，实施“双百千”工程，推进380个总投资1.5万亿元的重点项目，把国家应急产业基地打造成国内一流的产业园区。在构建交通新格局方面，建设城市轨道交通，建成龙烟铁路，完成大莱龙铁路扩能改造，争取潍莱高铁与济青高铁同步通车，推动尽早建设环渤海高铁，积极推进海青城铁，力争渤海海峡跨海通道、中韩铁路轮渡取得实质性突破，建成蓬栖、文莱、龙青高速公路。在强化要素新保障方面，实现海阳核电一期并网发电、二期启动建设，建成华电莱州电厂二期、中兴电力蓬莱电厂等重大能源项目，构建“双水源”“双气源”保障网络。在打造城市新亮点方面，开发烟台港一突堤，建成滨海西路及夹河桥，基本完成城市棚户区、危房、老旧小区改造。

决胜全面小康、率先走在前列，就要厚植优势、创立标杆。珍惜“烟台蓝”，着力保护好蓝色的天、蓝色的海，大力发展蓝色经济，持续提升蓝海实力和蓝色魅力，建成“本地人自豪、外地人向往”的宜商宜居宜游城市。提升“烟台造”，倡导“工匠精神”，推进“精致生产”，培育“烟台智造”，扩大烟台产品在汽车、高铁、海工、航空航天、核电等高端领域的应用，把烟台建设成为国内先进制造业的重要基地之一。做好“烟台人”，大力弘扬烟台精神、提升烟台价值、凝聚烟台力量，使“烟台好人”成为广大市民的共同追求，让全国文明城市成为永恒名片。

决胜全面小康、率先走在前列，就要补齐短板、整体提升。在三次产业中着力补齐服务业短板，依托制造业优势带动生产性服务业向专业化、价值链高端延伸，围绕挖掘消费潜力促进生活性服务业向精细化、高品质提升，以全面奔小康为引领推进公共服务业向便利化、高效率转变。到2020年，服务业增加值占比达到46%以上。在县域经济发展中着力补齐不平衡短板，开展新一轮县域经济提升行动，推动县城和重点镇发展成为新生中小城市，加大帮扶栖霞、长岛工作力度。在城乡发展中着力补齐农村短板，扎实做好“三农”工作，推动基础设施和公共服务向农村延伸。到2017年，基本完成农村危房改造；到2018年，实现农村无害化卫生厕所全覆盖。在经济社会发展中着力补齐社会事业短板，加大民生投入，增加公共服务供给，综合解决“生、老、病、养、学”等问题。在不同收入群体中着力补齐贫困人口短板，实施精准扶贫、精准脱贫，确保2017年6.9万贫困人口全部脱贫，让所有烟台人生活更有保障、更为公平，人生更有尊严、更加精彩。

决胜全面小康、率先走在前列，就要防范风险、坚守底线。坚守安全生产底线，健全“三二一”机制，促进安全生产实现根本性好转。坚守森林防火底线，确保防火责任落实到位、火源监控管理到位、突发火情处置到位。坚守生态环保底线，实行最严格的环境保护制度，构建“两屏两区八廊道”生态安全格局。坚守食药安全底线，健全产品追溯体系，严把从田间到餐桌的每一道防线，努力创建食品药品安全城市。坚守社会稳定底线，深入开展法治烟台、平安烟台、和谐烟台建设，争创全国社会治安综合治理优秀城市“七连冠”。坚守清正廉洁底线，始终保持良好作风，做到干部清正、政府清廉、政治清明。

各位代表，时序更替，梦想前行，决胜全面小康、率先走在前列是“十三五”的“烟台梦”。我们要凝聚全市人民的力量，全力推动生产总值向1万亿元迈进，一般公共预算收入向1000亿元迈进，固定资产投资突破8000亿元，规模以上工业主营业务收入突破2.5万亿元，高新技术产业产值突破1万亿元，城乡居民收入比2010年翻一番，全面提升产业品质、生活品质、生态品质、文化品质，努力把烟台建设成为经济竞争力更强、群众幸福感更足、环境友好度更高、文化影响力更大的成功城市。目标引领前进方向，目标凝聚奋斗力量。我们坚信，只要全市上下凝心聚力、攻坚克难，就一定能够跑好“十三五”这一棒，一定能够建成高质量的小康社会，一定能够实现“十三五”的“烟台梦”！

三、2016年的政府工作安排

今年是“十三五”的开局之年，是推进结构性改革的攻坚之年。我们要在战略上稳中求进，在战术上抓住关键，全力打好稳增长提质效的主动战，打好转方式调结构的持久战，打好改革开放创新的突破战，打好破解发展难题的攻坚战。主要预期目标是：全市生产总值增长8%左右，一般公共预算收入增长9.5%左右，固定资产投资增长13%左右，社会消费品零售总额增长11%左右，进出口保持适度增长，实际使用外资增长7%左右，城镇和农村居民人均可支配收入分别增长8%和8.5%左右。

(一)供需两端发力“稳增长”，重点推进供给侧结构性改革。在供给侧，调整优化投资、人才和环境政策，减少无效和低端供给，扩大有效和中高端供给，提高全要素生产率，为经济长期持续增长注入新动力。全面落实去产能、去库存、去杠杆、降成本、补短板“五大任务”。推进技术改造、兼并重组、转移合作，逐步化解钢铁、船舶、水泥等行业过剩产能。多措并举化解房地产库存，落实好住房公积金使用、信贷、税收等支持政策，有序推进“三个市民化”，吸引各类创业人才、年轻技能人才来烟购房、落户、兴业。完善全口径政府债务管理，推进债务结构调整和存量置换，防范债务风险。重点开展以降低企业成本为目标的制度性改革，减少企业交易、财务、电力、物流成本和税费负担。在需求侧，着力营造“能消费、敢消费、愿消费”的政策环境，积极培育新消费，拓展“老幼”两端消费，丰富健康和长期护理消费，发展文化娱乐消费，启动实施离境退税政策，激活境外消费者购物消费。发挥投资的关键作用，实施总投资7991亿元的200个市级重点项目。在市政公用设施、医疗、养老等领域推进PPP项目，鼓励社会资本、外商投资积极参与。发挥出口的促进作用，大力发展服务贸易，培育外贸综合服务企业，建设电子口岸和国际航运中心，推进保税港区二期封关运作，争取开通跨境电商保税进口业务和中韩海运邮路，有选择地进口能源资源性产品、关键设备、重要零部件和日用消费品。

(二)精准引导“调结构”，重点推进产业结构调整向产品层面延伸。继续强化产业行业分类指导，按照“五种类型”促进产业结构调整，深入实施“六升六降”产品升级方案，培育更具核心竞争力的优质产品集群。把工业结构调整作为壮大实体经济的重中之重。大力推进“两化”深度融合，发展智能制造、高端制造、绿色制造、精细制造。针对汽车、计算机、手机等33种产值过百亿元的工业产品，针对海工装备、机器人等高科技、高附加值产品，针对各类传统产品，整合资源分类扶持。实施品牌战略和标准化战略，鼓励企业主导或参与国际、国家、行业标准制修订，共建品牌联盟、抱团开拓市场。深化军民融合发展，拓展军民共用的先进技术、产业产品、基础设施。把服务业结构调整作为产业升级的重中之重。引进培育信息技术、现代物流等生产性服务业，丰富提升家政、健康等生活性服务业。加快大数据、云计算、物联网系统建设，抓好华为云数据中心等200个服务业重点项目。整合提升旅游资源，发展邮轮游艇经济，建设仙境海岸旅游带核心城市。剥离培育200家限额以上服务业企业。把农业结构调整作为带动广大农民奔小康的重中之重。着力推动农产品向特色、优质、生态、安全方向升级，扶持“三品一标”认证和国际认证，构建烟台特色优势产品价格指数体系。实施藏粮于地、藏粮于技战略，落实粮食安全保障措施，建设好42个国家级粮油高产创建万亩示范片。开展化肥农药使用量零增长行动。树立大食物理念，推进“海上粮仓”、海洋牧场建设。培强叫响苹果优势产区，打造全国苹果价格中心和交易中心。提升农业综合机械化水平。强化无规定动物疫病区运行管理。促进粮经饲统筹、农林牧渔结合、种养加一体化发展，培强做大农业龙头企业，规范发展农民合作社和家庭农场，全面提升农业社会化服务水平，大力发展农村电子商务，引导农村一二三产业融合发展。

(三)激发全社会活力“抓创新”，重点推进大众创业、万众创新。扶持发展“四新经济”，打造“四众模式”支撑平台，抓好“七个一批”市场主体培育，发展分享经济。完善以企业为主体、市场为导向、政府大力推动、产学研用相结合的紧密型创新体系，加强知识产权保护，发挥天使投资和成果转化引导基金作用，实施科技“创新券”制度，培育科技创新龙头企业和科技型中小企业。完善产业导航服务平台，加强科技企业孵化器建设，发展新型研发机构。健全人才流动、评价和激励机制，培养引进领军人才、青年骨干和优秀科研团队，加强企业家队伍建设。鼓励商业模式创新，引导工业企业由生产制造商向以制造为基础的综合服务商转型。发展平台经济，积极开展介于现货与期货之间大宗商品交易市场试点，拓展海洋产权交易中心和联合产权交易中心的功能。稳妥推进金融创新，做强法人金融机构，支持各类金融机构在我市开展业务，有序实施新型农村合作金融试点和互联网私募股权融资平台试点。充分利用多层次资本市场，促进企业多渠道融资。探索设立山东省区域性基金管理中心发展母基金，培育引进各类股权投资基金，加快打造全省金融板块“第三极”。扩大政策性农业保险品种与覆盖面。完善中小企业信用担保体系。加强地方金融监管，规范各类融资行为，严厉打击非法集资，坚决守住不发生区域性、系统性风险底线。

(四)内外联动“扩开放”，重点强化招商引资、优化生产力布局。坚持“大走访”常态化，更加精准高效地与世界500强及行业领军企业对接，深化与央企、大型民企、科研院所合作，拓展产业基金、外资并购、境外上市等多元化引资渠道，支持外资企业增资扩股，推动教育、文化、医疗、养老等服务业领域外资准入试点。加快建设中韩(烟台)产业园，重点推进智能制造、生命科学、金融服务等产业招商合作，探索对韩先行先试开放政策，扩大中韩优秀进出口企业互认范围，建设电子口岸国际贸易“单一窗口”，着力打造中韩自贸区建设前沿城市。深入挖掘与港澳台、日本经贸合作的潜力，加强与“一带一路”沿线国家、国际友城的经贸往来，着力打造“一带一路”战略支点城市。拓展与欧洲、美国、澳大利亚以及京沪深、环渤海区域的深度合作，密切与东亚城市间交流。支持本土企业根留烟台，建设跨国公司，拓展国际市场、开发境外资源、开展产能和装备合作、设立“海外仓”。发挥园区开放主阵地作用，创新园区体制机制，开展社会管理事务剥离和招商人员市场化试点。因地制宜建设国际学校、医院和社区，努力营造“亲商、爱商、安商、富商”的发展环境，使烟台成为本土企业发展的沃土、域外企业向往的热土。

(五)围绕深层次问题“促改革”，重点抓好上级改革方案落实和自主革新。搞好国家、省改革试点，探索可推广可复制政策、制度和措施。推进“先照后证”“证照分离”改革，实行收费清单管理，强化事中事后监管。有序发展混合所有制经济，建立以管资本为主的国资监管模式。完善预算管理体系，建立跨年度预算平衡机制，加大财政资金整合力度，推进税式支出管理改革。抓好农村集体资产确权到户和股份合作制改革，推动农村产权交易市场建设。实行不动产统一登记。推进机关事业单位养老保险制度改革，稳妥实施生产经营类事业单位转企改制，开展事业单位法人治理结构试点。实施公车改革，深化医药卫生、收入分配、价格、供销等各领域改革。积极推进具有较大牵引作用的本地改革措施，促进多重目标、多种政策、多项改革的平衡协调。

(六)坚持一体化发展“统城乡”，重点搞好基础设施建设和公共服务。尊重城市发展规律，坚持“五个统筹”，完善治理体系。高起点精准化规划提升城市品位。加快城市总体规划修编，编制近期建设、综合交通等各类规划，推进“多规合一”。提高规划设计水平，塑造具有历史文化底蕴、符合山海禀赋特色的城市风貌。高标准精品化建设打造城市经典。抓好年度投资601.5亿元的269个市区城建重点项目，启动城市轨道交通建设，实现胜利南路南延、城铁南站南路等道路竣工通车，推进慎礼等29个区片改造，实施标识规范提升等15项市政和绿化工程，完善慢行交通系统，推动城市停车产业化发展。启动“海绵城市”和地下综合管廊规划建设工作。加快铁路建设，龙烟铁路年内竣工，开工建设潍莱高铁，实施大莱龙铁路扩能改造工程。实现西港区至淄博输油管道、30万吨级原油码头试运行。加快建设3条高速公路。坚持最严格的耕地保护和节约集约用地制度，提高土地开发利用效益。高水平精细化管理保障城市运行。着力解决交通拥堵、乱搭乱建等各类“城市病”，提高老旧小区管理水平。抓好9项污水、垃圾处理及排水排污改造工程，用两年时间完成运行超过15年的供热和供水管网改造，新增供热面积100万平方米、天然气用户2万户。加强智慧城市建设，加快4G网升级改造、三网融合、光纤入户，扩大免费无线网络覆盖范围。抓好国家重点镇和省市示范镇建设。启动农村社区服务中心完善提升三年行动计划，加快建设县、乡、村互联互通的网络服务平台。推进农村亮化工程和取暖工程试点，改造农村厕所15万户，新建改造旅游厕所4420处。继续实施公路安全生命防护工程，改造13条普通国省干线公路，整治隐患道路2316公里，硬化农村公路360公里。抓好7座大中型水库除险加固工程，全面完成南水北调配套工程建设。

(七)严控严管“保生态”，重点实施“三清一绿”工程。实施清新空气工程，开展“五项整治”活动，健全大气污染联防联控机制。全市新建电动汽车快充站51个，在市区新上300辆纯电动公交车。实施清澈水源工程，严格执行《烟台市区饮用水水源地保护规划》，抓好47项水生态文明提升和10大类23项饮用水源地保护工程，确保如期实现水质提档升级目标。制定出台《烟台市落实<水污染防治行动计划>实施方案》，实施26条重点河流控制单元水质达标工程，水质稳定达标率达到90%以上。加大海洋生态环境保护修复力度。强化土壤、重金属、危险废物和化学品污染防治。实施清洁生产工程，严格执行排放新标准，启动燃煤机组超低排放改造，完成分散锅炉淘汰任务，鼓励使用清洁能源和环保新技术。实施绿色家园工程，加强景观通道、城郊森林、水源地绿化等生态建设，做好裸露山体恢复、森林和湿地保护工作，治理水土流失面积180平方公里，植树造林10万亩，共建共享天蓝、地绿、水清、景美的魅力烟台。

(八)以人为本“惠民生”，重点加大投资于人的力度。压缩“三公经费”和一般性开支，安排更多资金用于基本公共服务，确保经济下行压力不向民生传导。深入实施素质教育，大力发展职业教育，支持高等教育加快发展，扩大公益性和普惠性学前教育资源，推进“全面改薄”工程，逐步解决中小学“大班额”问题，改造校舍40万平方米以上。完成普通中小学校长职级制改革，

建设教育装备综合改革试验区。力争烟台格罗宁根大学实现暑期招生。积极创建“健康烟台”，推动医疗、医保、医药“三医联动”，完善基层医疗卫生服务体系，全面推开公立医院改革，加快建设烟台山医院新院等医疗机构。建立百岁老人长寿津贴发放市级配套制度，每千名老人拥有养老床位数达到36张以上。加快打造养老产业典范城市和医疗健康产业基地式城市。实施体育健康工程，发展赛事经济。大力弘扬社会主义核心价值观，深入实施“四德”工程。加强文化遗产保护，做好对胶东传统优秀文化特别是红色文化的持续挖掘和阐发。发展文化产业，加大文体场所设施的规划、建设和配备力度，创建国家公共文化服务体系示范区，繁荣群众文化生活。设立覆盖城乡的“书香烟台”建设指标评价体系。落实一对夫妇可生育两个孩子政策，提高出生人口素质。扎实推进史志档案、民族宗教、仲裁等工作。加强人民防空、国防教育、国防动员、民兵预备役和优抚安置等工作，争创新一届全国双拥模范城。

(九)强化保障“兜底线”，重点加强对特定人群特殊困难的精准帮扶。动员和聚集全社会力量，用心、用情、用力实施产业扶贫、教育扶贫、金融扶贫、行业扶贫，有序推进农村低保线与扶贫线“两线合一”，确保年内脱贫3.5万人。着力办好为民服务实事。进一步优化收入分配格局，不断缩小城乡居民收入差距。实施更加积极的就业创业政策，完善各项优惠措施，鼓励有志者特别是青年人创业，提升农民工职业技能，完善失业预防调控机制，落实援企稳岗政策，年内新增城镇就业13万人，农村劳动力转移就业2.2万人。启动全民参保登记，建立城镇职工大病保险制度，逐步提高养老金、失业保险金、城乡低保、农村五保等保障水平。发展慈善事业，及时开展临时困难救助。探索共有产权住房保障模式，新增发放租赁补贴1693户，改造棚户区1.23万户，货币化安置7000户，更好地改善群众居住条件。

(十)千方百计“增和谐”，重点强化安全稳定措施。深入推进“三城联创”。做好立法相关工作，实施“七五”普法规划，制定“法治地图”。完善立体化社会治安防控体系，加强禁毒工作，加大“天网”工程建设力度。全方位加强安全生产监管，排查整治安全隐患，坚决杜绝重特大事故、遏制较大事故、减少一般事故。强化应急管理，做好气象服务和防震、防汛、防台风及抗旱减灾、森林防火、海上搜救等工作。争创国家食品安全城市，在大中型超市和农贸市场建立快检室，建成7个县级病死畜禽集中处理场。完善基层民主自治制度，建立“四社联动”机制，实现政府治理和社会调节、居民自治良性互动。畅通诉求表达渠道，有效预防和化解社会矛盾。充分发挥工会、共青团、妇联等群团组织在社会治理中的重要作用。健全守信激励和失信惩戒机制，建设“信用烟台”。

四、进一步加强政府自身建设

面对新形势新任务，政府系统将不断巩固群众路线教育实践活动、“三严三实”专题教育成果，以严的纪律和实的作风强化执行力建设，确保干在实处、走在前列，全面兑现对人民的庄严承诺。

(一)推进依法行政，规范行政行为。政府系统将全面加强法治政府建设，严格按照法定权限和程序履行职责，坚持科学民主依法决策。严格执行市人大及其常委会决议决定，自觉接受人大的法律监督和政协的民主监督，扎实办理人大代表建议和政协提案。加大政务公开力度，及时回应社会关切。

(二)坚持执政为民，提升服务水平。政府系统将全面加强服务型政府建设，以简政放权为市场主体松绑，以降负减费为企业轻身，以“负面清单”划清政府与市场的界限，着力服务企业、服务基层、服务群众。强化“首接负责制”，再造工作流程，实现网上办事大厅和政务服务大厅协调互动，推进服务标准化、一体化，健全以服务对象满意为标准的政务服务监督、评价、考核机制，着力打造审批环节少、办事效率高、行政成本低的政务环境，让“亲民、爱民、亲商、爱商”体现在政府主动服务之中。

(三)强化责任担当，狠抓工作落实。政府系统将全面加强执行力建设，围绕率先走在前列的战略定位和“十三五”任务目标，定标明责狠抓落实。围绕“敢为、能为、有为”健全激励约束机制，为敢于负责者负责，为勇于担当者担当，让不作为、乱作为者无处立足、得到法纪惩处，让担当者、实干者有舞台、有作为、有地位。

(四)严守纪律规矩，树立廉洁形象。政府系统将全面加强作风建设，切实增强看齐意识，坚决贯彻执行党的路线方针政策。始终把纪律和规矩挺在前面，认真落实全面从严治党主体责任，自觉遵守《廉洁自律准则》《纪律处分条例》等规章制度。坚持高标准和守底线相结合，强化行政监察和审计监督，大力弘扬珍惜名节、注重操守、干净为官的优良传统，压缩消极腐败现象生存空间和滋生土壤，进一步营造风清气正的政务环境。

各位代表，时代赋予重托，实干铸就辉煌。让我们紧密团结在以习近平同志为总书记的党中央周围，在省委省政府和市委的坚强领导下，同心同德、真抓实干，顽强拼搏、开拓进取，为决胜全面小康、率先走在前列、赢得关键五年而努力奋斗！
